# Supplementary material for: Is weight-adjusted waist index more strongly associated with diabetes than body mass index and waist circumference?: Results from the database large community sample study
Source: PLoS One. 2024 Sep 26;19(9):e0309150. doi: 10.1371/journal.pone.0309150 (PMC11426486; doi:10.1371/journal.pone.0309150)
Supplement: Attachment — Submitted filename: Response to Reviewers.docx [file pone.0309150.s001.docx]

Dear Editor and Reviewers,

We appreciate the opportunity to allow us to revise our manuscript and thanks for the reviewers’ constructive comments and suggestions. We would like to submit our revised manuscript, entitled “Is weight-adjusted waist index more strongly associated with diabetes than body mass index and waist circumference?: results from the database large community sample study” for consideration for publication. In the revised manuscript, we have carefully addressed all comments and questions raised by reviewers point-by-point. We marked the changes in red in the revised paper. We greatly appreciate your time and efforts to improve our manuscript for publication.

Reply to Reviewers

Reviewer 1 #:

**Question 1:** While the title explicitly mention the comparators (BMI and WC), the abstract only describes WWI results. It would be good to add a sentence showing the association between DM and WWI was stronger than that between DM and WC and DM and BMI.

**Response:** Thank the reviewer for pointing out our oversight. The fact that WWI is more relevant than DM and WC has been added on lines 44-46.

**Question 2:** The main aim of the study comes too late in the introduction. The introduction of DM and its complication is a bit too long delaying the introduction of the main aim. The association/relationship between DM/related factors and WWI, WC and/or WC should appear early in the introduction.

**Response:** We are very sorry for the delay in the introduction of the main objective due to the cumbersome language. The introduction to DM has now been streamlined and the main purpose introduced in advance, in lines 56-68.

**Question 3**: The introduction also did not feature BMI and WC which are explicitly mentioned on the title. It looks like the aim was just to investigate WWI and not compare its performance/association against WC and BMI.

**Response:** Thanks to the reviewer for carefully pointing out our shortcomings, the introduction of BMI and WC has been added in lines 69-76 and marked in red font.

Reviewer 2 #:

**Question 1:**Abstract： Lines 21-23 paraphrase the sentences for readability

**Response:** Sorry, our expression is not clear and accurate enough. We sincerely thank the reviewer for your enthusiastic work. After a double check of grammar and word order, the content has been revised on lines 21-23.

**Question 2:** Materials and Methods: Exposure variable - Re-write the paragraph. It is not coherent.

**Response:** We think this is a good suggestion, and we apologize for our lack of clarity and accuracy. This has been amended in lines 137-146.

**Question 3:** What was the essence of only recording moderate physical activity?

**Response:** We greatly appreciate your professional comments on our articles. The questions for moderate activity in NHANES are as follows：In a typical week {do you/does SP} do any moderate-intensity sports, fitness, or recreational activities that cause a small increase in breathing or heart rate such as brisk walking, bicycling, swimming, or volleyball for at least 10 minutes continuously? There are fewer missing parts in this data, and the problems are more detailed and objective. In many similar cross-sectional studies, moderate activity also appears as a covariate[1–3].

**Question 4:** Results: Table 1–Amend the intervals used to generate quartiles e.g. value 11.22 is inclusive in Q2 and Q3.

**Response:** Thanks to the reviewer for pointing out our negligence carefully and accurately. We are very sorry and have corrected the relevant content in Table 1 by using open and closed intervals.

**Question 5:** Tables 2 and 3. The notes below the tables contain unnecessary definitions of abbreviations that are not used in the tables.

**Response:** Thank you for your careful examination. All abbreviations have been carefully reviewed and revised to ensure that each is necessary in the header or table, and the abbreviations in Tables 2 and 3 and their corresponding content are highlighted in red font.

**Question 6:** Discussion: The is no paragraph that would lead to answering the question featured in the title. Likewise, the discussion section should come up with possible explanations for the observed correlation between WWI and diabetes.

**Response:** Thank you very much for your comments on the discussion section. Our study shows that WWI is more strongly associated with diabetes than WC and BMI, and details similar studies on WWI. We have revised these contents in detail in lines 296-311 and 357-360 of the paper.

These comments are all valuable and enable us to greatly improve the quality of our manuscript. Our whole team made meticulous revisions to the manuscript word for word according to the reviewer's opinions, making the manuscript more fit with the title, the content more scientific and orderly, and the text expression clearer and smoother. We tried our best to improve the manuscript. These changes will not influence the content and framework of the paper. We appreciate for Editors/Reviewers’ warm work earnestly and hope that the corrections will meet with approval. Please do not hesitate to contact us with any further questions or recommendations.

Once again, thank you very much for your comments and suggestions.

Sincerely yours,

Jinli Guo

**References:**

1. Liu H, Ma Y, Shi L. Higher weight-adjusted waist index is associated with increased likelihood of kidney stones. Front Endocrinol. 2023;14: 1234440. doi:10.3389/fendo.2023.1234440

2.	Cao S, Hu X, Shao Y, Wang Y, Tang Y, Ren S, et al. Relationship between weight-adjusted-waist index and erectile dysfunction in the United State: results from NHANES 2001-2004. Front Endocrinol. 2023;14: 1128076. doi:10.3389/fendo.2023.1128076

3.	Wang K, Mao Y, Lu M, Liu X, Sun Y, Li Z, et al. Association between serum Klotho levels and the prevalence of diabetes among adults in the United States. Front Endocrinol. 2022;13: 1005553. doi:10.3389/fendo.2022.1005553
