# Supplementary material for: Is weight-adjusted waist index more strongly associated with diabetes than body mass index and waist circumference?: Results from the database large community sample study
Source: PLoS One. 2024 Sep 26;19(9):e0309150. doi: 10.1371/journal.pone.0309150 (PMC11426486; doi:10.1371/journal.pone.0309150)
Supplement: Attachment — Submitted filename: Response to Reviewers.docx [file pone.0309150.s002.docx]

Dear Editor and Reviewers,

We appreciate the opportunity to allow us to revise our manuscript and thanks for the reviewers’ constructive comments and suggestions. We would like to submit our revised manuscript, entitled “Is weight-adjusted waist index more strongly associated with diabetes than body mass index and waist circumference?: results from the database large community sample study” for consideration for publication. In the revised manuscript, we have carefully addressed all comments and questions raised by reviewers point-by-point. We marked the changes in red in the revised paper. We greatly appreciate your time and efforts to improve our manuscript for publication.

Reviewer #3: 1. Introduction

- The introduction is very long and unfocused, the authors should shorten it and clarify the message so it is clear to the reader what is the problem that the authors are addressing.

**Response:** We greatly appreciate your professional comments on our articles. Based on your comments, we have streamlined the introductory section by eliminating repetitive and irrelevant remarks. Starting from the severity of diabetic complications, we introduced the relationship between obesity and diabetes, transitioned to indices that can assess obesity, compared the advantages and disadvantages between WC, BMI, and WWI, and finally led to the purpose of our study.

- The authors make rather outdated statements like "BMI is the new norm for defining .....''

**Response:** Thank the reviewer for pointing out our oversight. In lines 68-70, we update the definition of BMI：BMI redefines “healthy weight” and “unhealthy weight” and is one of the most commonly used diagnostic tools. At the same time, more appropriate references were replaced.

- line 75 and 76 "WC is an essential part of the metabolic syndrome which also involves the detection of insulin resistance''. This sentence is confusing and should be clarified.

**Response:** Thanks to the reviewer for carefully pointing out our shortcomings. Because central obesity is closely related to metabolic diseases such as diabetes mellitus, hypertension, and cardiovascular and cerebrovascular diseases, and is an important contributing factor to the occurrence and development of metabolic syndrome, all of them have made central obesity as a necessary condition for the diagnosis of MS. WC can effectively respond to the degree of abdominal fat aggregation, and so it is used as a measure of central obesity[1]. In lines 74-76, we make this sentence more fluent and clear: WC plays an important role not only in the assessment of metabolic syndrome but also a central role in the detection of insulin resistance.

- Line 91 " associated with unhealthy body components..." it is body compartments not components

**Response:** Thank you for the linguistic details of our article. In line 91, replace "components" with " compartments".

- Line 100 "that the optimal BMI or the oldest elderly may be in the overweight or mildly obese range". This is also a very generalized and unsubstantiated statement.

**Response:** Sorry, our expression is not clear and accurate enough. In lines 97-99, we clarified the definition of the obesity paradox, where overweight and obese patients have a better prognosis than non-overweight or non-obese patients among those who suffer from cardiovascular disease[2].

- Line 112 "The correlation between WWI and diabetes has important clinical significance in promoting patients' physical and mental health, reducing social burden, and rationally allocating medical resources". It is unclear whether the authors are advocating for WWI as a predictor for diabetes or just correlated with diabetes. If it is the latter what is its significance then?

**Response:** Our study explored the correlation between WWI and diabetes and found that the correlation between WWI and diabetes was higher than the correlation between WC and BMI and diabetes. Our methodology was cross-sectional and could be used as a pilot study to guide future larger, prospectively randomised controlled studies.

2. Results

- Table 1: Did the authors stratify their study population tot ensure equal numbers among the four quartiles Q1- Q4 (N=1993). This was not indicated in their methods section.

**Response:** In lines 133-137, we go into detail in the methods section. In order to carry out the trend test, we used EmpowerStats software to quarter the study population according to the WWI interval and made the four groups nearly equal in size.

- Table 2: Model 3 N= 2120 while model 1 and 2 have n = 7973 participants. what happened to the missing participants in model 3?

**Response:** In order to analyze the relationship between WWI and diabetes in depth, we performed multivariate logistic regression analyses. Model 2 is adjusted for age, gender and ethnicity. To give stability to the results, we adjusted for confounders, which increased the number of covariates included in Model 3, including age, gender, race/ethnicity, educational level, HDL-C, LDL-C, triglyceride, total cholesterol, Family PIR, total cholesterol, marital status, hypertension status, moderate activity, protein, total sugars, total fat, smoking status and drinking alcohol status. In Model 3, the number of people decreased because more confounding factors were included, resulting in missing variables. In order to ensure accuracy, the corresponding participants were reduced.

- Figure 2 - 5. There figure legends are just the covariates assessed and not a description of the figures. The figures also lack any key and can not be interpreted.

**Response:** We have made changes to the legends. To test for nonlinear relationships, we performed smoothed curve fitting for WWI and diabetes. Fig 2 is the fit of the linear regression between WWI and diabetes under the fully adjusted model. Horizontal coordinates represent WWI, vertical coordinates represent diabetes. The solid red line indicates the smoothed curve fit between the variables, and the blue bands indicate the 95% confidence intervals of the fit. Fig 3 is the result of the smoothed curve fit with diabetes under the fully adjusted model after transforming WWI from a continuous variable to a quartile categorical variable. And deleted redundant fig 4 and fig 5.

3. Discussion

- The authors do not really discuss their results in relation to other published literature, the discussion section needs to be re-written. The discussion is also unfocused and delves into topics that are outside the topic of the manuscript (Lines 312 - 353)

**Response:** Thank you very much for your comments on the discussion section. In lines 271-253, We have rewritten the discussion section in detail. The relationship between the results and other published literature is insightfully discussed based on your comments, and have highlighted the main points of the discussion[3–9].

These comments are all valuable and enable us to greatly improve the quality of our manuscript. Our whole team made meticulous revisions to the manuscript word for word according to the reviewer's opinions, making the manuscript more fit with the title, the content more scientific and orderly, and the text expression clearer and smoother. We tried our best to improve the manuscript. These changes will not influence the content and framework of the paper. We appreciate for Editors/Reviewers’ warm work earnestly and hope that the corrections will meet with approval. Please do not hesitate to contact us with any further questions or recommendations.

Once again, thank you very much for your comments and suggestions.

Sincerely yours,

Jinli Guo

**References**

1.	Tian J, Qiu M, Li Y, Zhang X, Wang H, Sun S, et al. Contribution of birth weight and adult waist circumference to cardiovascular disease risk in a longitudinal study. Sci Rep. 2017;7: 9768. doi:10.1038/s41598-017-10176-6

2.	Lavie CJ, Milani RV, Ventura HO. Obesity and cardiovascular disease: risk factor, paradox, and impact of weight loss. J Am Coll Cardiol. 2009;53: 1925–1932. doi:10.1016/j.jacc.2008.12.068

3.	Xu G, Liu B, Sun Y, Du Y, Snetselaar LG, Hu FB, et al. Prevalence of diagnosed type 1 and type 2 diabetes among US adults in 2016 and 2017: population based study. BMJ. 2018;362: k1497. doi:10.1136/bmj.k1497

4.	Haire-Joshu D, Glasgow RE, Tibbs TL. Smoking and diabetes. Diabetes Care. 1999;22: 1887–1898. doi:10.2337/diacare.22.11.1887

5.	Yan Y, Wu T, Zhang M, Li C, Liu Q, Li F. Prevalence, awareness and control of type 2 diabetes mellitus and risk factors in Chinese elderly population. BMC Public Health. 2022;22: 1382. doi:10.1186/s12889-022-13759-9

6.	Resnick HE, Valsania P, Halter JB, Lin X. Differential effects of BMI on diabetes risk among black and white Americans. Diabetes Care. 1998;21: 1828–1835. doi:10.2337/diacare.21.11.1828

7.	Nakagami T, Qiao Q, Carstensen B, Nhr-Hansen C, Hu G, Tuomilehto J, et al. Age, body mass index and Type 2 diabetes-associations modified by ethnicity. Diabetologia. 2003;46: 1063–1070. doi:10.1007/s00125-003-1158-9

8.	Abate N, Chandalia M. The impact of ethnicity on type 2 diabetes. J Diabetes Complications. 2003;17: 39–58. doi:10.1016/s1056-8727(02)00190-3

9.	Haffner SM, Mitchell BD, Hazuda HP, Stern MP. Greater influence of central distribution of adipose tissue on incidence of non-insulin-dependent diabetes in women than men. Am J Clin Nutr. 1991;53: 1312–1317. doi:10.1093/ajcn/53.5.1312
