# Supplementary material for: Is weight-adjusted waist index more strongly associated with diabetes than body mass index and waist circumference?: Results from the database large community sample study
Source: PLoS One. 2024 Sep 26;19(9):e0309150. doi: 10.1371/journal.pone.0309150 (PMC11426486; doi:10.1371/journal.pone.0309150)
Supplement: Attachment — Submitted filename: Response to Reviewers.docx [file pone.0309150.s003.docx]

Dear Editor and Reviewers,

We appreciate the opportunity to allow us to revise our manuscript and thanks for the reviewers’ constructive comments and suggestions. We would like to submit our revised manuscript, entitled “Is weight-adjusted waist index more strongly associated with diabetes than body mass index and waist circumference?: results from the database large community sample study” for consideration for publication. In the revised manuscript, we have carefully addressed all comments and questions raised by reviewers point-by-point. We marked the changes in red in the revised paper. We greatly appreciate your time and efforts to improve our manuscript for publication.

Reviewer #3:  Results:
In Table 1 the authors report to have compared the categorial variables using chi squared test. This is not correct as this test is used to compare only two categorical variables. ANOVA would be a better statistical method.
**Response:** We thank the reviewer for the valuable suggestion on the article at the statistical level, you are correct in your suggestion. In our study, the weighted chi-square test was used for the comparison between categorical variables, and for the comparison between continuous variables, ANOVA was used. In fact, our study did follow the statistical methodology described above. We apologize for this misunderstanding due to an oversight in our presentation. In Table 1, we restate this part.

-In the linear regression analysis, the authors have now removed the number of participants (N = ), why is this the case?
**Response:** Thank you for your meticulous review. After a unanimous consultation with our team, and taking into account the published literature[1–3], it was felt that Tables 2 and 3 contained too much redundant information, so the number of participants was deleted and a uniform statement was made in the Statistical Methods section.

- The discussion is still unfocused and the authors do not really discuss the implications of their results in relation to current practice.

**Response:** Thank you very much for your comments on the Discussion section. Our team reviewed the content of the Discussion section and removed some non-diabetes related statements (e.g., hypertension, hyperlipidemia, cardiovascular disease, and cerebrovascular disease in lines 305-307), as well as removing content not related to preventing the onset of diabetes in lines 315-317, allowing for more focused content. A previous study of diabetes prediction methods was added to lines 323-333 of the manuscript and compared to our study, discussing the implications of our findings for current practice.

These comments are all valuable and enable us to greatly improve the quality of our manuscript. Our whole team made meticulous revisions to the manuscript word for word according to the reviewer's opinions, making the manuscript more fit with the title, the content more scientific and orderly, and the text expression clearer and smoother. We tried our best to improve the manuscript. These changes will not influence the content and framework of the paper. We appreciate for Editors/Reviewers’ warm work earnestly and hope that the corrections will meet with approval. Please do not hesitate to contact us with any further questions or recommendations.

Once again, thank you very much for your comments and suggestions.

Sincerely yours,

Jinli Guo

**References**

1.	Liu Z, Kuo P-L, Horvath S, Crimmins E, Ferrucci L, Levine M. A new aging measure captures morbidity and mortality risk across diverse subpopulations from NHANES IV: A cohort study. PLoS Med. 2018;15: e1002718. doi:10.1371/journal.pmed.1002718

2.	Zhang Y, Wu H, Li C, Liu C, Liu M, Liu X, et al. Associations between weight-adjusted waist index and bone mineral density: results of a nationwide survey. BMC Endocr Disord. 2023;23: 162. doi:10.1186/s12902-023-01418-y

3.	Qin Y, Qiao Y, Wang D, Li M, Yang Z, Li L, et al. Visceral adiposity index is positively associated with fasting plasma glucose: a cross-sectional study from National Health and Nutrition Examination Survey 2017-2020. BMC Public Health. 2023;23: 313. doi:10.1186/s12889-023-15231-8
